# Supplementary material for: Chromothripsis during telomere crisis is independent of NHEJ, and consistent with a replicative origin
Source: Genome Res. 2019 May;29(5):737–49. doi: 10.1101/gr.240705.118 (PMC6499312; doi:10.1101/gr.240705.118)
Supplement: Supplemental Material [file supp_gr.240705.118_Supplemental_file_1.zip › contigs/annotated_contigs/DB50/contig.2.DB50_length_531_mean_cov_10.1148775895.docx]

**DB50_length_531_mean_cov_10.1148775895**

```
AGCTGCTGCTTCCAGGACCCTAAGGGGTGTGGAATCTGAGTGACTCTCTCTCCTTCCCTGGCCTTTTGCTTTGGGATTTGCCCGTGGGG
 >chr10:126690162-126690399 + E=5e-131
CAGCCCCACCCCACTGCGTCCTGACCCCTGTGAGTGTACGGGCAGCTCCCTGCTCGCCCGGCTCCCCAGCCCTGCTCCTGACACCAAGA

CCCACCCAGTGCTTCCGGTACCTGGGTGCTGCCCCGACTGGCCGTGAAGGATTCAACTA|TAACGAGCAGCCAGAGTTGAGATGGCTAG
                                                             >chr10:126552832-126553126
TCTAAGCCTCTAGTCATTCAAATGTTTAGCCTGTTGAGGCTGGAAGAGCTCCAGAGACCCTCCGTCTGGCCCTCATCTTACACGGGGAG
+ E=2e-165
ACTGAGGTCTGCGAGGAGCCCGTGGGCTGGGAATGACAGTCTCTGTTTCCTGCCCCGCCTAGGACCTGAGACCACATTCCTTTTTTAGA

GCACAGCGTGGCAGTGAGCCAGCATCTCAGCCCCAAAACAGGTCAGGCCAGGGAGGAGTCCAGGCCTGGTGACCATAGCTGCTGTCA
```
